# Daily variation of macronutrient concentrations in mature human milk over 3 weeks

**DOI:** 10.1038/s41598-021-89460-5

**Published:** 2021-05-13

**Authors:** Gabriela E. Leghi, Ching T. Lai, Ardra Narayanan, Merryn J. Netting, Michael Dymock, Alethea Rea, Mary E. Wlodek, Donna T. Geddes, Beverly S. Muhlhausler

**Affiliations:** 1 School of Agriculture, Food and Wine, The University of Adelaide, Adelaide, Australia; 2 School of Molecular Sciences, The University of Western Australia, Perth, Australia; 3 Women and Kids Theme, South Australian Health and Medical Research Institute (SAHMRI), Adelaide, Australia; 4 Centre for Applied Statistics, University of Western Australia, Perth, Australia; 5 Mathematics and Statistics, Murdoch University, Perth, Australia; 6 Department of Physiology, School of Biomedical Sciences, The University of Melbourne, Melbourne, Australia; 7 Commonwealth Scientific and Industrial Research Organisation (CSIRO), Adelaide, Australia

**Keywords:** Paediatric research, Nutrition

## Abstract

Human milk (HM) composition is known to be highly variable, both between individuals and across the duration of lactation. It is less clear, however, to what extent fat, lactose and protein concentrations in HM change daily over shorter time periods in mature HM, and no studies have evaluated this to date. The aim of this study was to systematically assess and compare HM macronutrient concentrations in samples collected at different times of day, from left and right breasts and daily across a 3-week period in the same woman. Fifteen lactating women (1.6–4.9 months postpartum) collected daily pre-feed HM samples from both breasts each morning for 21 consecutive days and completed intensive sampling once a week (morning, afternoon and evening samples) during this period. Concentrations of fat, protein and lactose in HM did not differ according to time of day, day of week or breast used for collection. The results of this study suggest that pre-feed samples collected at any point across a 3-week period and from either the left or right breast provide comparable measures of fat, protein and lactose concentrations in mature HM, in pragmatic studies where women are collecting their own HM samples.

**Clinical trial registration**: Australian New Zealand Clinical Trials Registry (ACTRN12619000606189).

## Introduction

Human milk (HM) composition is highly variable, both between individuals and across lactation^1–3^. Fat concentration is generally considered to be the most dynamic, and varies across a single breastfeed or expression, as well as across the day, in the majority of studies^2,4,5^. Protein and lactose concentrations are generally reported to be more stable, both across a feed and over the course of the day, however not all studies have produced consistent results^2,4,6^. Considerable shifts in macronutrient (protein, fat, lactose) composition have also been reported to occur across lactation, particularly in the first few weeks postpartum^1,7^. It is less clear, however, to what extent fat, lactose and protein concentrations in HM change daily over shorter time periods in mature HM, and no studies have evaluated this to date.

There have been suggestions that there are differences in the composition of HM sampled from different breasts in the same woman^8^. This is potentially due to differences in breast fullness and time since the infant last fed from that specific breast, since both of these factors have been associated with differences in fat content^5^. However, evaluation of between-breast differences in HM macronutrient composition is limited and data in the existing literature are not consistent. Three studies have found no significant differences in HM concentrations of fat, protein or lactose^9^ or in casein and whey proteins^10^ between breasts over a 24-h period or over a 3 week period (sampling HM once a week)^8^. One of the studies did, however, identify significant differences between breasts in HM protein content in 5 of the 20 participants^8^.

The dynamic variations in HM macronutrient composition, as well as potential differences in composition between breasts, suggest that the method used to collect the sample has the potential to have a significant impact on the results obtained from analyses of macronutrient composition. Our recent systematic review of collection methods used in studies of HM macronutrient composition revealed the use of a broad range of different sampling methodologies^11^. However, very few studies had directly compared the impact of the different collection methods on fat, protein and lactose concentrations. As a result, the extent to which factors such as the time of day, day of the week and/or collection breast can impact HM composition, and therefore need to be factored in when designing studies investigating HM composition, remains unclear. This information is particularly important for studies undertaken in the community, in which women collect their own HM samples, since there is less capacity to precisely control the time of day and time relative to feeds that samples are collected. In addition, since collection of repeated samples increases participant burden, it is important to establish whether collection of multiple samples is necessary, or whether it is possible to obtain representative information on HM composition in an individual woman using a less frequent sampling regimen.

Therefore, the primary aim of this study was to use the opportunity presented by a clinical study involving regular collection of a large number of HM samples from breastfeeding women over a 3-week period to systematically evaluate the impact of day of collection during the week, time of day of collection and collection breast on HM macronutrient concentrations.

## Methods

### Study design

This study utilised samples collected from women who were participating in an open label dietary intervention trial which aimed to determine the effect of improving dietary quality in lactating women for 2 weeks on the composition of their HM. Women completed a 1 week baseline period, during which they consumed their habitual diet. Following the baseline week, women completed a 2-week dietary intervention phase targeted towards reducing fat and sugar intake, during which they were provided with all meals and snacks. Informed written consent was obtained from all participants for this study. All research was performed in accordance with relevant guidelines and regulations. This study was approved by the Human Research Ethics Committee of The University of Western Australia (RA/4/20/4953) and registered with the Australian New Zealand Clinical Trials Registry (ACTRN12619000606189) on 23/04/2019.

### Study participants

Women were approached to participate in the study through community centres and existing networks. Women were eligible to participate in the study if they were exclusively breastfeeding a term singleton infant who was between 6 and 20 weeks postpartum at the time of the first study visit and growing normally (according to WHO standards)^12^. Women and infant pairs were excluded from participation if there were any known major infant congenital abnormalities or health issues that could affect feeding behaviour, pregnancy complications (e.g. gestational diabetes, preeclampsia, fetal growth restriction), maternal diabetes, maternal diseases known to affect gastric absorption, restrictive diets (e.g. gluten free, dairy free, milk free, vegan) or maternal smoking.

### Human milk sample collection

Women were instructed to collect daily HM samples (~ 5 mL) each morning before the first morning feed and at least 2 h after the previous feed every day throughout the 3-week study. In addition to this, they were instructed to undertake intensive milk sampling once a week throughout the study. This involved collecting 3 HM samples (from both breasts in separate vials which were analysed separately): one before the first feed in the morning, one in the afternoon and one in the evening for one 24-h period. Women were permitted to select the day of the week that they undertook the intensive sampling and the exact times that samples were collected and were instructed to keep this consistent across the 3 weeks of the study. If sampling was completed as per the study protocol, the total number of HM samples collected across the study period for each woman was 54 samples (27 from each breast). At each time point, women were asked to obtain pre-feed samples from each breast after first wiping their hands and breasts with an anti-bacterial wipe (to ensure aseptic collection) either by hand expression or using a breast pump. Participants stored the samples in their home freezer until weekly collection by study staff. Samples were transported in cooler bags with ice to maintain low temperature until reaching the biochemical lab where samples were stored at − 80 °C for later analysis.

### Sample preparation

All samples were thawed for 2 h at room temperature, mixed on Intelli-Mixer (RM-2, ELMI, Ltd, Riga, Latvia) using UU mode for 15 s at 50 rpm and after 3 gentle top-to-bottom inversions, aliquoted into 1.5 mL microtubes (Sarstedt, Numbrecht, Germany) prior to analysis. Whole milk was used to measure fat concentrations and HM samples were defatted for measurement of protein and lactose concentrations. The standard assays were carried out using a JANUS automatic pipetting workstation (PerkinElmer, Inc., Waltham, MA, USA) and measured on a EnSpire plate reader (PerkinElmer, Inc., Waltham, MA, USA).

### Macronutrient measurements

Fat concentration of milk samples was measured by a modified and validated creamatocrit method^13,14^. Briefly, samples were mixed with an Intelli-mixer (RM-2, ELMI Ltd, Riga, Latvia) using the UU mode for 15 s at 50 rpm and followed by 3 gentle top-to-bottom inversions. The mixed milk was sampled in duplicate using glass capillary-tubes (41A2502, Kimble-Chase, USA), sealed at one end with tube sealing compound (43510, Kimble-Chase, USA) and spun in a flat-bed centrifuge (CEN 96221, Phoenix Scientific Industries Ltd, USA) designed for capillary-tubes for 10 min at 12,000×*g*. Spun capillary-tubes were read on Creamatocrit Plus device (Medela, AG) to determine the fat content. The Creamatocrit device provided fat content as a percentage, which was then converted to total fat content (g/L) using a validated equation^15^. The effective creamatocrit reading range was 1.1% to 50.9%, as reported by the manufacturer (Medela, AG). Our research group has previously reported that the results obtained using this Creamatocrit approach are strongly correlated with results obtained using the reference gravimetric method^14^. The read capillary-tubes were then cut and the skim milk portion was retained for the protein and lactose assays.

Protein concentration in the milk sample was measured in duplicate by a modified Bradford method using a commercial protein reagent (5000006, Bio-Rad Laboratories, USA)^16^. Protein standards were prepared in the lab from an aliquot of HM and the protein concentration determined by the Kjeldahl method, as described by Atwood and Hartmann (1992)^17^ and Mitoulas et al. (2002)^2^. The recovery of a 1.03 g/L human milk protein standard (prepared in-house) added to the milk samples was 101 ± 2.1% (n = 6). The detection limit of this assay was 0.03 g/L, the inter-assay CV was 18% and the intra-assay CV was < 10% (n = 22).

Lactose concentration in the milk sample was determined in duplicate by an enzymatic method^2,18^. The enzymes used in the assay, β-galactosidase (10105031001, Roche), glucose oxidase type II (G6125) and peroxidase from horseradish type II (P8250), were purchased from Sigma-Aldrich, Australia. The recovery of 250 mM lactose standard (made from α-lactose monohydrate, Sigma-Aldrich, Australia) added to the milk samples was 99.3 ± 1.6% (n = 6). The detection limit of this assay was 6 g/L, the inter-assay CV was 10% and the intra-assay CV was < 10% (n = 22).

### Statistical analysis

The data was analysed by fitting a linear mixed effects model to each of the response variables: fat concentration, protein concentration and lactose concentration. Day (categorical), breast (indicator) and time of day (categorical) were input as explanatory variables for each of the models. The analyses were conducted for each week separately to detect “within week” differences, which tested for differences across the days (1–7), breast (L/R) and time of day (morning/afternoon/evening). In all models, the mothers were fitted as random effects so as to account for the within mother variation. Model selection was carried out using backwards selection. All statistical analysis, including descriptive statistics, was undertaken using the statistical programming language R^19^ (software version R.3.5.2). A *p* value of < 0.05 was considered statistically significant.

## Results

### Study participants

Of the 66 women currently breastfeeding or expressing HM who initially expressed interest in participating and requested further information, 38 confirmed their interest and completed the eligibility assessment. Of the 18 who were eligible and enrolled in the study, 15 women completed the study. Clinical and sociodemographic characteristics of the study participants (mothers and infants) are shown in Table 1. Study participants were predominantly of Caucasian background (n = 13), with one woman identifying as Hispanic and one not providing information on ethnicity. The majority of participants had completed a University degree (n = 12). The age of the infants at the time of study entry ranged from 1.6 to 4.9 months, and there were approximately equal numbers of males and females.

**Table 1:** Demographic characteristics of participating mothers and infants^a^.

Characteristics	Mean ± SD	Range
**Mothers**		
Age (years)	32 ± 3	27–37
Pre-pregnancy weight^a^ (kg)	69.4 ± 10.8	55.0–89.0
Pre-pregnancy BMI^a^ (kg/m^2^)	25.1 ± 4.1	17.2–32.7
Current weight (kg)	71.4 ± 10.3	54.4–80.7
Current BMI (kg/m^2^)	25.8 ± 4.1	17.1–33.0
**Infants**		
Age (months)	3.1 ± 0.8	1.6–4.9
Sex^a^ (M/F)	8/6	–
Birth weight^a^ (kg)	3.6 ± 0.4	2.9–4.3
Birth length^a^ (cm)	51.1 ± 1.7	48–53
Current weight (kg)	6.1 ± 1.0	4.6–7.6
Current length (cm)	60.3 ± 3.3	55.2–65.5

*BMI* body mass index, *F* female, *M* male, *SD* standard deviation.

^a^Missing information from one participant.

A total of 796 HM samples were provided by the 15 participants and analysed in duplicate for each of the macronutrients (Table 2). Eleven women provided all 54 HM samples and the remaining 4 women provided between 47 and 53 HM samples.

**Table 2:** Samples analysed for macronutrient concentrations in human milk^a^.

Weeks	Samples per woman	Total samples, *n*
1	17–19	270
2	16–18	263
3	12–20	263
Total		796

Breast		Samples, *n*
Left	24–28	400
Right	23–27	396

^a^N, number of samples.

### HM macronutrient concentrations across days of the week and between breasts

**Figure 1:**
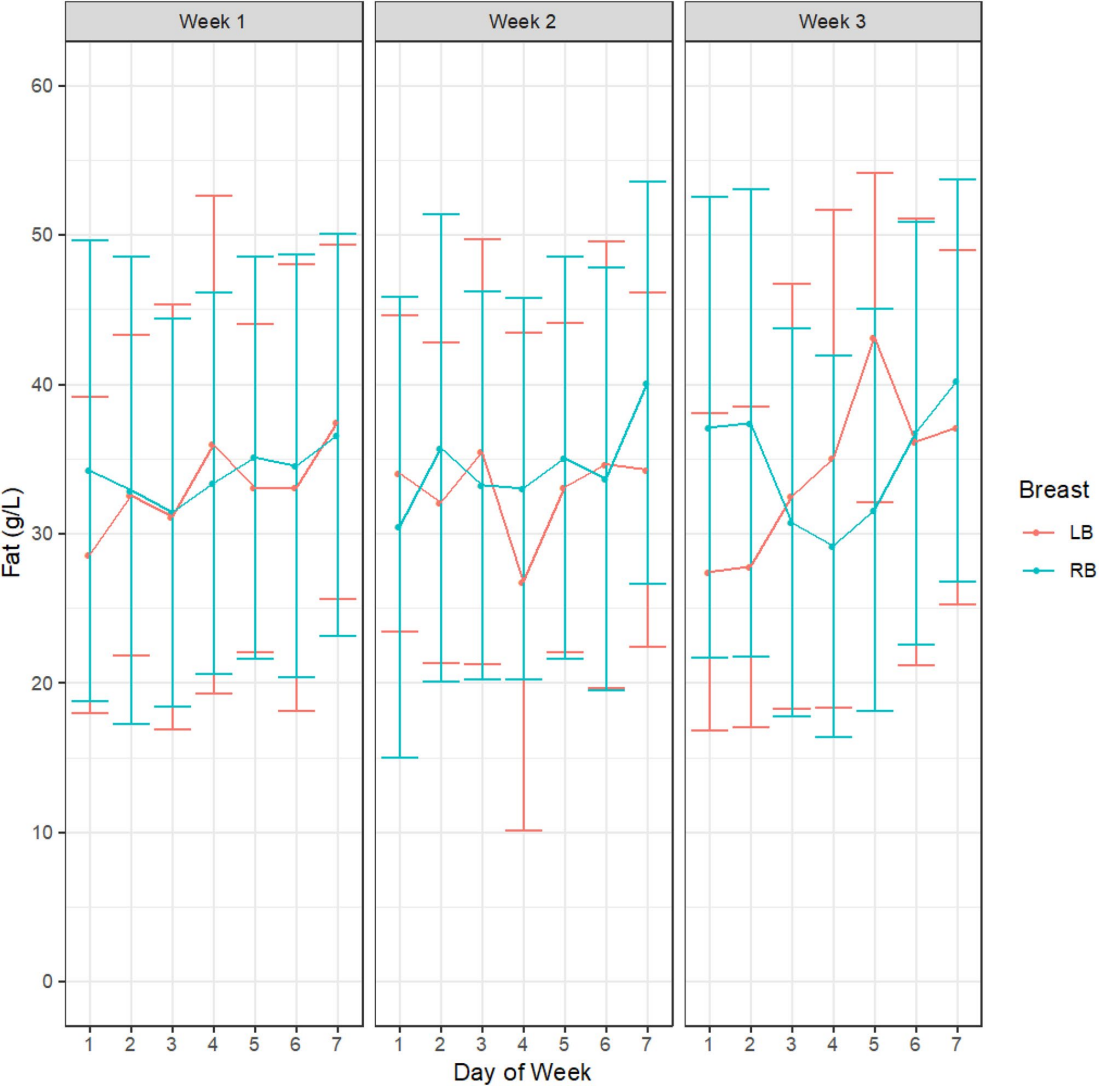
HM fat concentrations within participants across the days and between breasts for week 1, week 2 and week 3. Left breast (LB, red); Right breast (RB blue). Values are shown as mean and standard deviation.

**Figure 2:**
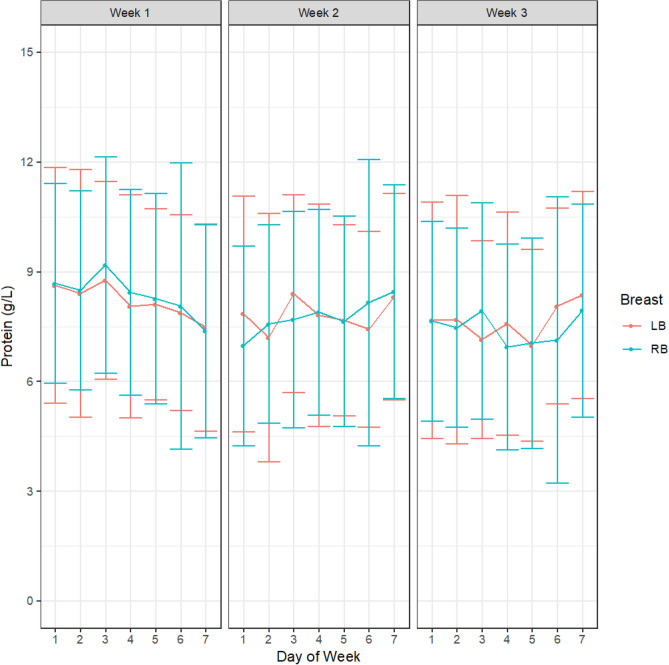
HM protein concentrations within participants across the days and between breasts for week 1, week 2 and week 3. Left breast (LB, red); Right breast (RB blue). Values are shown as mean and standard deviation.

**Figure 3:**
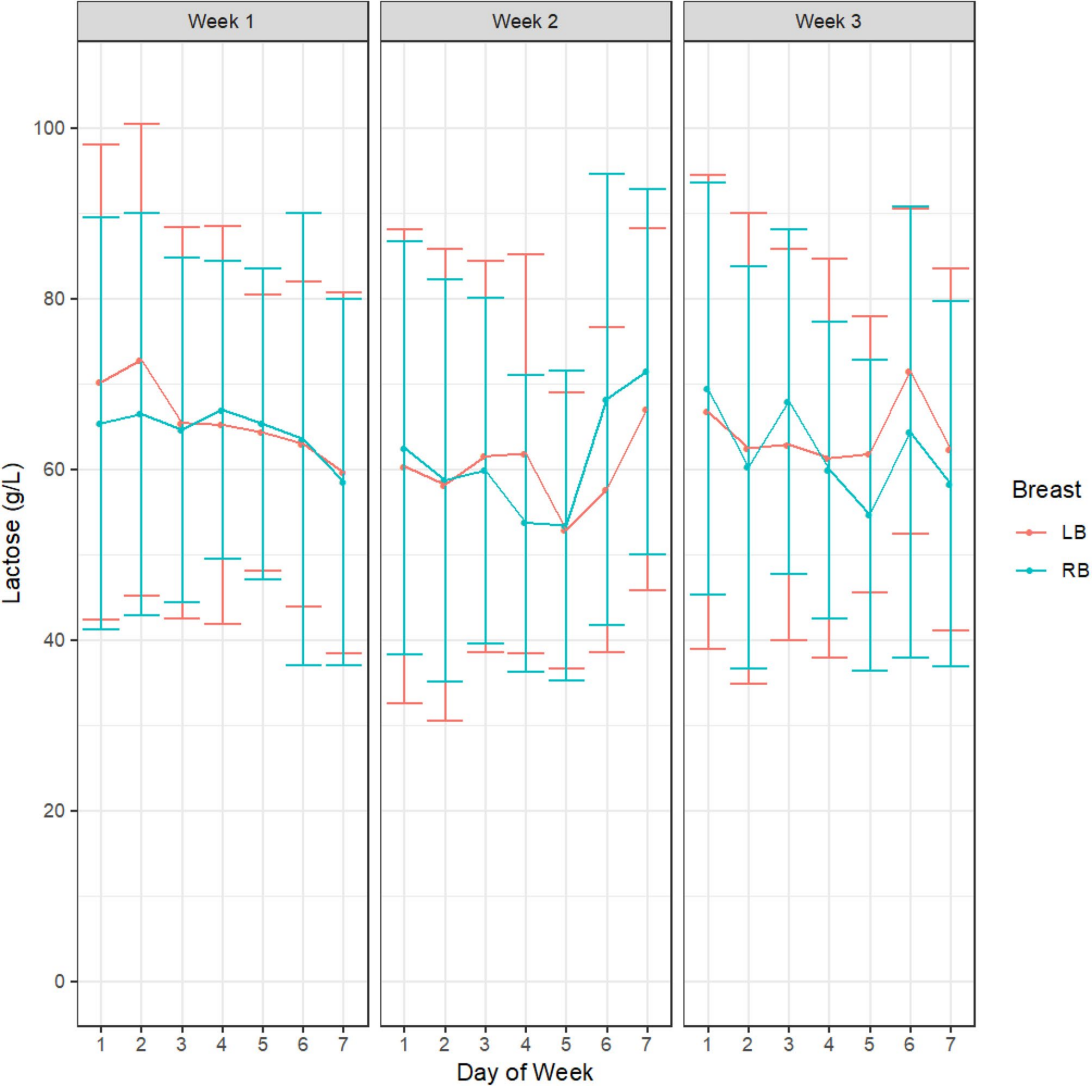
HM lactose concentrations in HM within participants across the days and between breasts for week 1, week 2 and week 3. Left breast (LB, red); Right breast (RB blue). Values are shown as mean and standard deviation.

There were no differences in HM concentration of either fat (week 1, *p* = 0.34; week 2, *p* = 0.27; week 3, *p* = 0.15, Fig. 1), protein (week 1, *p* = 0.37; week 2, *p* = 0.36; week 3, *p* = 0.97, Fig. 2) or lactose (week 1, *p* = 0.51; week 2, *p* = 0.19; week 3, *p* = 0.11, Fig. 3) either between different study days in any of the 3 study weeks or across the 3-week study period. There were also no differences in concentrations of either fat (week 1, *p* = 0.51; week 2, *p* = 0.35 week 3, *p* = 0.95), protein (week 1, *p* = 0.62; week 2, *p* = 0.96; week 3, *p* = 0.23) or lactose (week 1, *p* = 0.40; week 2, *p* = 0.25; week 3, *p* = 0.15) between right and left breasts at any point in the study. The samples analysed included all samples collected in the study (from both daily and intensive sampling).

### Impact of time of day of collection on HM macronutrient concentrations

There were no significant differences in the concentrations of protein and lactose in HM between samples collected in the morning, afternoon and evening in any of the study weeks (Table 3). The fat content was not different throughout the day with the exception of week 2 of the study, where fat content was significantly higher in HM samples collected in the afternoon compared to those collected in the morning or evening (*p* = 0.001) (Table 3).

**Table 3:** Mean fat, protein and lactose concentrations in HM collected at different times of day across the 3 study weeks^a^.

	Morning	Afternoon	Evening	*p* values
**Fat (g/L)**				
Week 1	33.26 ± 14.10	36.85 ± 11.35	33.24 ± 7.70	0.350
Week 2	32.55 ± 14.93	43.43 ± 20.24	34.39 ± 13.18	**0.001**
Week 3	34.02 ± 13.70	39.00 ± 15.06	35.83 ± 13.50	0.280
**Protein (g/L)**				
Week 1	8.24 ± 3.08	8.19 ± 2.92	8.32 ± 1.63	0.964
Week 2	7.82 ± 2.62	7.74 ± 1.90	7.83 ± 2.19	0.942
Week 3	7.56 ± 2.52	7.38 ± 3.19	7.42 ± 2.49	0.811
**Lactose (g/L)**				
Week 1	64.89 ± 21.82	68.88 ± 26.04	62.77 ± 22.03	0.408
Week 2	60.69 ± 20.53	61.13 ± 19.23	61.85 ± 20.36	0.927
Week 3	64.06 ± 23.00	61.32 ± 26.43	58.47 ± 19.86	0.051

^a^Values are shown as mean ± standard deviation.

A *p* value of **<0.05** was considered statistically significant.

## Discussion

Given the dynamic nature of HM composition, questions have been raised as to which sampling regimens appropriately account for this variation and thus provide reliable measures of HM composition. In the current study, in which women collected pre-feed samples daily for 3 weeks and three times daily for one day each week, we did not identify any significant differences in concentrations of any macronutrients in HM according to either the day or time of collection or the collection breast. These results suggest that it is not necessary to sample mature milk repeatedly in exclusively breastfeeding women in order to obtain reliable measures of HM composition. However, in studies where the primary aim is to assess the intake of specific macronutrients in the infant, the inclusion of additional samples, collected post- as well as pre-feed, and measuring the volume of milk consumed, is also important.

As previously reported, there is considerable variation between women in HM macronutrient composition^6,7,20^. However, what is less understood are the acute temporal changes in HM composition within an individual and, therefore, how frequently it is necessary to collect HM samples in order to obtain a reliable estimate of the composition of an individual’s milk. In the current study, we found macronutrient concentrations in HM to be remarkably consistent in pre-feed HM samples that were self-collected from the same woman daily across a 3-week period, with no differences in the HM concentrations of fat, protein or lactose during this time. While no previous studies to our knowledge have sampled this intensively, our results are consistent with a prior study that reported no differences in HM fat, protein and lactose concentrations between HM samples collected from the same individual once a week for 3 weeks^8^. Our findings have relevance for longitudinal studies, since they provide confidence that less frequent sampling (weekly or less) is appropriate for obtaining reliable estimates of mature HM composition in an individual woman. In the case of population studies, these findings support the suggestion that a HM sample collected from a woman at a single time point in lactation can provide a reasonably reliable estimate of her milk composition, at least in the relatively short term (weeks). Therefore, this approach would be appropriate as a basis for estimating average composition in a population. It is important to note, however, that all women in this sample were between ~ 2 and 5 months postpartum, and thus producing mature HM, and were exclusively HM feeding, and more substantial changes across time are likely to be observed earlier postpartum, when women are producing colostrum or transitional milk^7,21^ and at weaning, when composition changes again^22–24^.

Time of day of sample collection has also been suggested to have an influence on HM composition^4,25^. We found no difference in mean protein or lactose content between samples collected at different times of day, which is consistent with previous reports^4,10^ and supports the absence of significant daily variability in the levels of these macronutrients in human milk. Interestingly, and in contrast to a number of previous studies that have reported lower fat concentrations in HM samples collected at night, compared to earlier in the day^26,27^, we found no consistent difference in fat content of HM across the day over the 3 study weeks, with the exception of the afternoon sample at week 2. It is important to note that HM fat concentrations from these samples were within reported normal ranges for mature HM across the first 6 months (27–32 g/L), including a coefficient of variation of 37.3%^28^, and across different collection methods (30.6–44.6 g/L)^11^. This is different to another study conducted by our group, George and colleagues (2020), which suggested that fat content was lower in HM samples collected in the morning in comparison to those collected in the afternoon and evening^29^. In this other study, however, the time of sample collection in relation to time since the previous feed was much more tightly controlled than in the present study^29^. Since the fat content of HM is strongly related to the fullness of the breast and time since last feed^26^, this difference may well account for this result. Overall, our findings suggest that the time of HM collection relative to infant feeding, infant feeding pattern and/or breast fullness at the time of collection are likely to be more important determinants of HM fat content than the time of day of collection per se.

Differences in HM concentrations in samples collected from the left and right breasts in the same woman at the same time point have been reported in a small number of prior studies^2,8,9^, which would suggest that it is important to consistently sample from the same breast over time. Consistent with the majority of previous studies, however, we found no differences in concentrations of fat, protein or lactose in HM collected daily from right and left breasts at the same time point^2,8,9^. This is also in line with data suggesting that breast dominance (the most frequently fed from), which typically does vary between breasts, does not appear to affect HM macronutrient concentrations^9^, although it has been associated with differences in milk production^2,26,30^. It therefore appears that other factors, including breast fullness and time since last feed, which are consistently reported to affect HM fat content, are more important factors to consider in a sampling protocol, rather than specifically needing to distinguish between right and left breasts.

A major strength of this study is the availability of both daily HM samples as well as samples collected at different times of day across a 3-week period. This enabled us to assess daily, within-day and between-breasts changes over time, and the collection of a large number of samples (~ 50 samples per participant and > 750 samples in total) for the assessment of macronutrient composition. Further, no women in the study sample had conditions, including diabetes and gestational diabetes or were smokers, which have been shown to impact milk composition^31,32^. This is important, given that all these conditions have the potential to not only impact on overall HM composition, but could also potentially affect how composition changes over time and could therefore have confounded the results. The major limitation to the current research is the fact that all samples were collected prior to feeding, which means that we were not able to use these data to directly compare the composition of pre-feed, post-feed or pooled HM samples. As indicated above, the primary objective was to assess changes in HM composition in the same woman over time and required women to collect a large number of samples over a short time period, thus pre-feed collection was a pragmatic and appropriate choice. When the aim of the study is to obtain an estimate of what the infant is actually consuming, however, a different sampling approach, including collection of pre- and post-feed samples over a 24-h period and measures of milk production would be required^11^.

## Conclusion

In conclusion, our findings indicate that the pragmatic approach of collecting pre-feed samples at one time point across a 3-week period provides a reliable estimate of HM composition in exclusively breastfeeding women between 1.6 and 4.9 months postpartum, irrespective of the breast of collection. These findings are important for the design of future studies to investigate HM composition, and would tend to suggest that there is limited need for repeated sampling of women across a week in order to obtain a reliable estimate of the macronutrient content of her mature breast milk. The results also imply that other factors, such as infant feeding pattern, time since last feed and breast fullness, are likely to be more important determinants of HM fat content than time of day of collection, and challenge the current dogma that fat content increases progressively across the course of the day. Further studies which directly investigate the key drivers of the variation in fat content of HM are warranted.
